# Effects of methanolic mealworm extract on zebrafish: growth, immunity, and gene expression

**DOI:** 10.1186/s12917-025-05051-w

**Published:** 2025-10-14

**Authors:** Mansoureh Abdolmanafi, Roghieh Safari, Seyed Hossein Hoseinifar, Laura Gasco, Metin Yazici

**Affiliations:** 1 Department of Fisheries, Faculty of Fisheries and Environmental Sciences, Gorgan University of Agricultural Sciences and Natural Resources, Gorgan, Iran; 2 Faculty of Marine Sciences and Technology, Iskenderun Technical University, Iskenderun, Hatay Türkiye; 3 Department of Agricultural, Forest and Food Sciences, University of Turin, Torino, 10124 Italy

**Keywords:** Innovative ingredients, Zebrafish model, Immune modulation, Insect meal, Aquaculture nutrition

## Abstract

This study evaluated the effects of methanolic mealworm extract (MME) dietary supplementation on growth performance, immunity, biochemical markers, histomorphology, antioxidant status, and gene expression in zebrafish (*Danio rerio*). Four groups were fed diets containing 0% (MME0, control), 0.25% (MME1), 0.5% (MME2), and 1% (MME3) MME for 8 weeks. Growth performance improved significantly in a dose-dependent manner, with the MME3 group showing the highest weight gain (+ 80%, *p* < 0.05), feed efficiency, and survival rate. MME2 and MME3 significantly increased mucosal immune parameters, including total protein, lysozyme, and alkaline phosphatase (ALP) activity (*p* < 0.05). The levels of total protein, immunoglobulin, and lysozyme in the serum also increased significantly in the MME2 and MME3 groups. Glucose levels decreased in a dose-dependent manner (*p* < 0.05), whereas cholesterol, albumin, triglycerides, and LDH levels remained unchanged (*p* > 0.05). Histological analysis of the intestinal tissue revealed significantly greater villus height in the MME2 group and villus diameter in the MME3 group (*p* < 0.05), with no significant differences in the other groups (*p* > 0.05). Antioxidant enzyme activities (SOD and CAT) and their gene expression did not differ significantly among groups (*p* > 0.05). Liver enzyme analysis revealed increased ALP levels in the MME2 and MME3 groups (*p* < 0.05), whereas ALT and AST levels remained unchanged (*p* > 0.05). Methanolic mealworm extract significantly increased the expression of immune-related genes in zebrafish (*p* < 0.05). The highest *LYZ* expression was observed in the MME3 group (3.92-fold), with no difference between MME1 and MME2 (*p* > 0.05). *TNF* levels were highest in MME2 (4.51-fold) and MME3 (5.50-fold). *IL-1β* expression showed a dose-dependent increase, with the MME3 group (3.91-fold) displaying the highest level (*p* < 0.05). Growth-related genes, including *GH*, *IGF*, and appetite-related *GHRL*, were significantly elevated in the MME2 and MME3 groups (*p* < 0.05). In conclusion, MME, particularly at 1%, significantly enhanced zebrafish growth, immunity, and gene expression, suggesting its potential for broader aquaculture applications after being tested in other fish models.

## Introduction

Fish meal has served as a crucial protein source for the aquaculture feed sector owing to its high protein content, superior amino acid composition, and exceptional digestibility. Furthermore, the increasing aquaculture activities to meet the protein needs of the growing population have necessitated a significant supply of feed [24, 25]. Primary feed components such as protein and lipid are primarily sourced from fish meal and fish oil from fish caught from natural stocks [17, 34]. As a consequence of the overexploitation of wild fish stocks, decreasing availability of fish meal, and increasing prices, research into alternative feed sources in aquaculture and efforts to reduce the industry’s dependency on fish meal and fish oil have gained importance [13, 28]. Some of these alternative sources can be used as substitute products, while others can be utilised as functional feed additives [4, 5].

Aquaculture expands to address growing demand, with a shift towards intensive systems that carry the potential for immune suppression and outbreaks of disease [40]. The excessive use of antibiotics in aquaculture has resulted in significant concerns regarding food safety, compromised immunity, and the rise of bacterial resistance [22]. Therefore, there has been an increased interest in novel natural feed ingredients that can reduce or even replace chemotherapies [39]. Functional feed additives, which have been widely researched in aquaculture to determine their effectiveness, include probiotics [15, 38], prebiotics [26], medicinal and aromatic compounds [29], herbal products [16], essential oils [36, 42], organic acids [27], and algal products [23, 42]. In recent years, insect meal has emerged as a popular alternative to fish meal and oil [12, 18, 25].

Insects are increasingly recognized as promising alternative protein sources for both freshwater and marine fish due to their nutritional richness in amino acids, lipids, vitamins, and minerals [11]. Compared to conventional livestock, insect farming is more environmentally sustainable, offering advantages such as lower greenhouse gas emissions, efficient bioconversion of organic waste, and reduced land and water usage [31]. Positive results have also been obtained in studies investigating insect meals as potential substitutes for fish meal and fish oil in aquafeeds [3, 14, 21]. Among insect species, the yellow mealworm (*Tenebrio molitor*) is authorized for use in aquafeeds under European Union regulations and is one of the most extensively studied for partial or full fish meal replacement [1, 10].

Mealworms are easily cultivable organisms that can be raised using a range of agricultural and other low-quality organic materials, thereby minimizing environmental impact. Additionally, their nutritional composition is readily adjustable [10, 19]. Larvae and pupae of mealworms exhibit notable protein levels (47–63%) and lipid (31–41%) content. Nonetheless, their amino acid profile and fatty acid compositions may not fully meet the nutritional demand of aquatic animals [10, 34].

Numerous studies have investigated the incorporation of *Tenebrio molitor* as a fish meal alternative in aquafeeds, reporting beneficial effects on growth, nutrient utilization, immunity, antioxidant capacity, and organ health in various species, including gibel carp (*Carassius gibelio*, CAS V) [43], largemouth bass (*Micropterus salmoides*) [6, 9] and European seabass (*Dicentrarchus labrax*) [2]. While inclusion levels varied across studies, partial or full replacement of fish meal with *T. molitor* generally yielded positive physiological responses without adverse effects on performance or metabolism.

However, despite these advantages, the direct use of raw insect meals still presents several limitations that challenge their broad application in aquafeeds. The nutritional composition of insect meals can vary significantly depending on the substrate used for rearing, processing methods and developmental stages. Their high lipid content may interfere with feed stability and formulation, and their large-scale application is often considered economically unfeasible [10]. To address these limitations, extract-based approaches have gained growing interest, aiming to concentrate bioactive compounds such as antioxidants, antimicrobial peptides, and immunostimulants, while minimizing unwanted components like excess fat. Among various solvents, methanol is widely used due to its high efficiency in extracting phenolics, flavonoids, and other polar compounds with proven biological activities (Monteiro et al., 2021). Nevertheless, the application of methanolic extracts derived from T. molitor in aquaculture remains largely unexplored, and their potential effects on fish growth, immunity, and gene expression have not been fully elucidated.

In this context, zebrafish (*Danio rerio*) represents a well-established and cost-effective model for nutritional studies in aquaculture [23, 30]. Its short life cycle, small size, and well-characterized genome enable high-throughput in vivo trials and facilitate the use of advanced molecular tools such as transcriptomic and genotyping analyses (Ulloa et al., 2014). These characteristics make zebrafish particularly suitable for investigating diet-induced physiological and molecular responses. Despite growing interest in insect-based feed ingredients, there is still a significant gap in understanding the impacts of insect-derived extracts, particularly methanolic preparations, on fish biology at both systemic and molecular levels.

Accordingly, the present study was designed to evaluate the effects of dietary methanolic yellow mealworm extract (MME) on zebrafish, focusing on growth performance, skin mucus and serum immunity, antioxidant enzyme activities, intestinal histomorphology, and the expression of genes related to immunity, oxidative stress, and metabolism.

## Materials and methods

### Fish supply and culture conditions

In the study, 600 individuals of 20-day-old zebrafish with an average weight of 0.15 ± 0.01 g utilized. The fish were sourced from the Gorgan Mahi Ornamental Fish Centre located in Golestan province and carefully transported to the aquaculture laboratory of Shahid Naser Fazli Barabadi at Gorgan University of Agricultural Science and Natural Resources. After being acclimated to the experimental conditions for ten days, the fish were randomly distributed in 12 aquariums (60 L), with a density of 50 fish larvae per aquarium, for four different treatment groups (MME0, MME1, MME2, MME3). Three replicates were conducted for each treatment group. The fish were fed with control feed (Biomar, France) up to satiation during the adaptation period.

### The preparation of the mealworm extract, diets and feeding trials

Due to its superior ability to extract polar compounds, yield higher concentrations of targets, enable a faster extraction process, and facilitate easier downstream processing, we utilize methanol as the extraction solvent at a ratio of 3:1 (solvent to sample). In the first stage of the process, 100 g of dried mealworm larvae provided by Varjavand Hirkan Co. was ground into powder using a grinder. This powder was then mixed with 300 mL of methanol (98%) and left to incubate at 25 °C for 72 h. Afterwards, it was filtered through Whatman filter paper. The yielding filtered extract was dried using a freeze-dryer to obtain the mealworm methanolic extract (MME). Experimental diets were prepared by supplementing varying levels (0.25%, 0.5%, and 1%) of MME into commercial feed (Biomar fish food). Table 1 provides, approximate composition of the diet and dry mealworm powder. During this procedure, the MME supplements were sprayed on the commercial diet, mixed manually, and then each experimental diet was coated with 3% Gelatin. To avoid the possible effects of Gelatin, the control diet was also coated with 3% gelatin. All the diets were dried in a clean place at room temperature (25 °C). To prevent oxidation, the diet was prepared weekly and stored in a refrigerator at 4 °C until use. The prepared treatment groups with MME were assigned specific codes as follows: MME0 (0%, control); MME1 (0.25%); MME2 (0.5%); MME3 (1%). Throughout the 8-week trial period, the fish were fed up 4% body weight, 4 times daily. During the trial period, water quality parameters in the tank were measured daily, and water temperature, dissolved oxygen, and pH were maintained and monitored daily at 25 ± 2 ◦C, 7.9 ± 0.1 mg/L and 7 ± 0.2.

**Table 1:** Approximate composition of the commercial diet and dry mealworm powder

Approximate composition of diet (as % of Dry matter)	
Dry matter	92.22
Crude protein	36.7
Crude lipid	11.13
Ash	3.48
Approximate composition of powdered dry mealworms (%)	
Protein	40.13
Lipid	20.72
Fiber	16.81
Ash	6.71
Moisture	5.35

### Evaluation of growth performance

Growth performance parameters were calculated using the standard formulas provided below. To measure the weight of the fish, all fish were deprived of food for 24 h, anesthetized with clove powder (250 mg L^−1^), and then weights were measured (using a digital scale) and lengths were recorded using callipers.

$$\mathrm{Weight}\;\mathrm{gain}\;(\mathrm{WG},\;\mathrm g)\:=\:\mathrm{final}\;\mathrm{weight}\;(\mathrm{FW})\:-\:\mathrm{initial}\;\mathrm{weight}\;(\mathrm{IW});$$ 

$$\mathrm{Weight}\;\mathrm{gain}\;(\mathrm{WG},\;\%)\:=\:100\times\;(\mathrm{FW}-\mathrm{IW})/\mathrm{IW}.$$ 

$$\begin{aligned} &\mathrm{Specific}\;\mathrm{growth}\;\mathrm{rate}\;(\mathrm{SGR},\;\%)\:=\:100\;\times\;(\ln\;\mathrm{FW}\:-\:\ln\;\mathrm{IW})/\\&\mathrm{number}\;\mathrm{of}\;\mathrm{trial}\;\mathrm{days}; \end{aligned}$$ 

$$\mathrm{Feed}\;\mathrm{conversion}\;\mathrm{ratio}\;(\mathrm{FCR})\:=\:\mathrm{Quantity}\;\mathrm{of}\;\mathrm{feed}\;\mathrm{provided}\;(\mathrm{dry}\;\mathrm{weight})/\mathrm{WG};$$ 

$$\mathrm{Survival}\;\mathrm{rate}\;(\mathrm{SR},\;\%)\;=\;(\mathrm{final}\;\mathrm{fish}\;\mathrm{count}/\mathrm{initial}\;\mathrm{fish}\;\mathrm{count})\;\times\;100.$$ 

$$\mathrm{Condition}\;\mathrm{Factor}\;(\mathrm{CF})\:=\:100\;\ast\;(\mathrm W/\mathrm L3).$$ 

$$\mathrm{Feed}\;\mathrm{Efficiency}\;(\%)\;=\;(\mathrm{Weight}\;\mathrm{gain}/\mathrm{Feed}\;\mathrm{intake})\;\mathrm x\;100.$$ 

### Measurement of skin mucus and serum immuno-biochemical parameters

#### Sampling of skin mucus

Sampling was conducted following a minor modified version of the method described by Vakili et al. [37]. At the end of the 60-day feeding trial, each treatment group comprised nine randomly selected and fasted fish for 24 h, which were individually placed in a polyethene bag with 10 ml of 50 mM NaCl solution (Sigma, Steinheim, Germany), and the fish were Gently rubbed for about 30 s. Following this, mucus samples pooled from each container were gathered into 10-ml tubes, then subjected to centrifugation at 1500 × g for 10 min at 4 °C, and finally preserved at −80 °C until enzyme analysis.

### Measurement of the investigated SM immune parameters

The skin mucus (SM) total Ig content was quantified following the method outlined by Rouhani et al. [30]. Briefly, the total protein concentration of mucus samples was assessed using a 12% solution of polyethylene glycol (Sigma) before and after precipitation of the immunoglobulin molecules. The variation in protein levels was quantified as the total Ig content of the skin mucus.

The SM lysozyme activity in the samples was determined using the turbidimetric method assay, employing the lysozyme-sensitive Gram-positive bacterium *Micrococcus luteus* (Sigma), following the protocol outlined in previous studies [32]. An equivalent volume of mucus sample (50 µL) was combined with lyophilized *Micrococcus luteus* (Sigma) suspension in a 96-well plate, followed by an incubation period of 15 min at 30 °C, during which absorbance readings were taken over a 50-minute duration. The enzymatic activity was quantified as the amount of enzyme required to reduce the absorbance by 0.001 min^−1^ at 450 nm.

The alkaline phosphatase activity was assessed following the protocol outlined by [35]. Briefly, A mixture of alkaline buffer solution (Sigma-Aldrich) (1 ml) and fish homogenate samples (20 µl) was incubated at 37 °C for 5 min. To stop the reaction process, NaOH solution (0.05 N) was introduced into the mixture. The final absorbance was measured at 410 nm using a UNICO UV-2100 PC spectrophotometer (UNICO, Beijing, China).

The total protein concentrations were assessed utilizing the biuret method with a commercially available kit from Pars Azmoon Co., Iran. The recommended procedure provided by the manufacturer was adhered to for the quantification of total protein levels.

### Sampling of whole body serum

At the conclusion of the experiment, three fish per tank were anesthetized with clove powder (0.5 g L^−1^) and euthanized with care in accordance with ethical guidelines. Subsequently, head and caudal fins of fish were removed, and they were promptly immersed in liquid nitrogen for preservation at −80 °C. The samples were homogenized in PBS and subsequently centrifuged at 3000 × g for 10 min at 4 °C to obtain the supernatant. The extracted whole-body serum was then stored at −80 °C until further analysis.

### Serum biochemical measurements

Serum total Ig and total protein level, and lysozyme activities were measured similar to the measurements conducted on skin mucus. Commercial laboratory kits (Zellbio, Germany) were used to determine Total cholesterol, Glucose, Albumin, Triglycerides and LDH levels in serum, in accordance with the manufacturer’s guidelines [23].

### Intestinal histology

To analyze the structure of the intestines, the method described by Yazici et al. [42] was utilized with some minor modifications. Briefly, at the end of the experiment, the anterior portion of the intestines from three fish in each group was collected. For the fixation of the tissues, they were placed in a 10% (v/v) neutral buffered formalin solution for one day before being transferred to 70% ethanol. Samples were handled with an automated tissue processor, which involved dehydrating them in various concentrations of ethanol, clearing them with xylene, and embedding them in paraffin wax. The tissue samples were sliced into thin sections of 4–5 μm thickness using a microtome from Leica Biosystems in the USA and stained with hematoxylin and eosin (H and E). The slides were observed and captured with a light microscope (BX51 Olympus, Tokyo, Japan). Morphological measurements were taken by assessing the length and width of the villi, as well as the thickness of the muscular layer, in five randomly chosen areas per fish.

### Analysis of antioxidant activity

Sampling was conducted as described by Yan et al. [41]. Briefly, Liver tissues were obtained from 9 fish in each treatment group. After sampling, fish Livers were homogenized in 50 mM ice-cold potassium phosphate buffer (1:8, w/v, pH 7.0). The resulting homogenate solution was then centrifuged at 10,000 rpm and 4 °C for 10 min, and the resulting supernatant was separated. Subsequently, the supernatants were measured for antioxidant enzyme activities such as SOD and CAT using commercial kits (Zellbio, Germany)(Mahmoudi et al., [23].

### Assay of liver enzymes

The activities of alanine aminotransferase (ALT), aspartate aminotransferase (AST), and alkaline phosphatase (ALP) in liver homogenates were assessed through the utilization of commercially available kits from Pars Azmoon Co., located in Tehran, Iran. The analysis was conducted in accordance with the procedures outlined by Moss and Henderson (1999). The outcomes were quantified as U g^−1^ protein. Protein concentrations were assessed utilizing a total protein assay kit from Pars Azmun Co., Iran, and the results were reported as grams per deciliter [37].

### Evaluation of gene expression

#### Sampling

Following the the 8-week feeding trial, the Livers, brains, and intestines of 9 fish randomly selected from each treatment group were carefully dissected for the expression of genes related to immunity (*LYZ*,* IL-1B*,* and TNF-α*), antioxidants (*SOD* and *CAT*), growth (*GH* and *IGF-1*), and appetite (*GHRL*) after euthanasia with an overdose of anesthesia (500 mg L^−1^ clove solution). Subsequently, the tissue samples were rapidly frozen in liquid nitrogen and preserved at −80 °C for subsequent analysis [23].

#### Isolating total RNA

Total RNA was extracted from 50 mg of tissue utilising the Esterabad-Zistfan-Pishro-Azma RNA extraction kit in accordance with the manufacturer’s instructions. To eliminate genomic DNA contamination, the RNA underwent DNase I treatment (Fermentas, Lithuania) following the manufacturer’s guidelines. The concentration of RNA samples was determined by measuring absorbance at 260/280 nm using a Nanophotometer (IMPLEN-P100), while the integrity was confirmed via ethidium bromide staining of 28 S and 18 S ribosomal RNA (rRNA) bands on a non-denaturing agarose gel (1.5%).

#### cDNA synthesis

cDNA synthesis was conducted using a cDNA synthesis kit (GentiBio, Korea) following the protocol. Assessment of synthesized cDNA was conducted using conventional PCR prior to real-time PCR to validate the primer accuracy and cDNA synthesis [32].

Table 2 displays the primer sequences employed for detecting gene expression across different categories, such as immune (*LYZ*,* IL-1B*,* TNF-α*), antioxidant (*SOD* and *CAT*), growth (*GH* and *IGF-1*), and appetite-related (*GHRL*) genes. The primer sequences were designed using Primer3 software based on GenBank sequences. The quantification of target gene expression levels was normalized by utilizing the β-actin gene as an internal control.

**Table 2:** The primer sequences used for real-time PCR for the selected genes involved in immunity, antioxidant activity, growth regulation, and appetite in zebrafish

Primer	Sequences	Annealing (C◦)	Primer efficiency	Accession number	Sample
IL-1b q-PCRF IL-1b q-PCRR	CGTCTCCACATCTCGTACTCA GTGTCTTTCCTGTCCATCTCC	59	95%	AY340959.1	Intestine
TNF-a q-PCRF TNF-a q-PCRR	CTGCTTCACGCTCCATAAGA CTGGTCCTGGTCATCTCTCC	59	95%	AY427649.1	Intestine
LYZ q-PCRF LYZ q-PCRR	GGCAGTGGTGTTTTTGTGTC CGTAGTCCTTCCCCGTATCA	59	95%	NM_139180.1	Intestine
SOD q-PCRF SOD q-PCRR	GGGTGGCAATGAGGAAAG GCCCACATAGAAATGCACAG	59	95%	BC055516.1	Liver
CAT q-PCRF CAT q-PCRR	GCATGTTGGAAAGACGACAC GTGGATGAAAGACGGAGACA	59	95%	AJ007505.1	Liver
GH q-PCRF GH q-PCRR	CTGCTTCACGCTCCATAAGA CTGGTCCTGGTCATCTCTCC	59	95%	AJ937858.1	Brain
IGF1 q-PCRF IGF1 q-PCRR	GGCAGTGGTGTTTTTGTGTC CGTAGTCCTTCCCCGTATCA	59	95%	NM131825.2	Liver
GHRL q-PCRF GHRLq-PCRR	ATGTTTCTGCTCCTGTGTGT GCTTCTCTTCTGCCCACTCT	59	95%	EU908736.1	Intestine
β- actin q-PCRF β- actin q-PCRR	AGCAGATGTGGATCAGCAAG TACCTCCCTTTGCCAGTTTC	59	95%	NM131031.1	Brain, Liver, Intestine

### Quantitative real-time PCR

Quantitative real-time polymerase chain reaction (qPCR) was carried out utilizing an iCycler instrument manufactured by Bio-Rad in the United States and Fermentas Maxima SYBR Green qPCR Master Mix (Fermentas), along with Gene-specific primers. The thermal cycling program consisted of an initial denaturation phase at 94 °C for 5 min, followed by 40 cycles of denaturation at 94 °C for 10 s, annealing at 59 °C for 10 s, and extension at 72 °C for 10 s. Each reaction was executed in triplicate as detailed in Table 2. The standard curve generated from serial dilutions of the standard sample showed a linear relationship between the log of the concentration and the Ct values, with a slope of (−3.02) - (−3.4) and an *R*² value of aproximatly 0.95, This indicates efficient amplification with a high degree of linearity, confirming the reliability of the qPCR assay for quantification. Non-significant differences of Ct values among treatment groups and small SD (less than 1) suggested that β-actin is stable and suitable for normalization. The fold change in the relative expression of immune response (*LYZ*,* IL-1b*,* TNF-α*), antioxidant (*SOD* and *CAT*), growth (*GH*, *IGF-1*) and appetite (*GHRL*) was determined using the 2^^(−ΔΔCt)^ method, where ^ΔCt^ represents the difference between the cycle threshold (Ct) values of the treated and control samples. Data analysis was conducted using Bio-Rad System software, version 2.00 (Hercules, CA, USA). The experiment included nine biological replicates and three technical replicates [37].

### Statistical analyses

This experiment was conducted using a completely randomized design. 2^−ΔΔCt^ method, a standard quantitative PCR (qPCR) method used for comparing gene expression levels between samples by calculating the difference in threshold cycle (Ct) values normalized to a reference gene. Non-significant differences of Ct values among treatment groups and small SD (less than 1) suggested that β-actin is stable and suitable for normalization. Along with data related to growth, antioxidant activity, whole-body extracts, and mucus immune factors, were tested for normality using the Kolmogorov-Smirnov test and for homogeneity of variances. The data were analyzed using one-way ANOVA, followed by Tukey’s post hoc test. Results are presented as mean ± standard deviation (X ± SD). SPSS software (version 16) and GraphPad Prism were used for data analysis.

## Results

### Growth performance

Table 3 presents the growth performance observed in zebrafish fed diets enriched with MME supplementation. The addition of MME positively influenced the growth performance parameters of zebrafish. With increasing dosage, a rise was observed in all examined parameters. Although there was a trend of increasing weight gain with dosage, a statistical significance (*p* < 0.05) was observed only in the MME3 group, with a significant increase of 80%. Length gain, condition factor and survival rate were higher in the MME2 group compared to MME0 and MME1 (*p* < 0.05), while the MME3 group was significantly different from all other treatments (*p* < 0.05). A significant improvement in feed conversion ratio was observed in the MME2 and MME3 groups compared to MME0 and MME1 (*p* < 0.05), with the best performance observed in MME3. Although there was no significant difference in FCR between the MME1 and control groups, higher inclusion levels (i.e., MME2 and MME3) significantly decreased FCR compared with the control group (*p* < 0.05). Specific growth rate, feed efficiency and survival rate were higher in all mealworm extract (MME) groups than in the control (*p* < 0.05), with the highest values observed in MME3.

**Table 3:** Growth performances, survival and feed efficiency of zebrafish fed diets with various levels of methanolic *T. molitor* extract (MME) for 8 weeks (*n* = 50)

	MME0	MME1	MME2	MME3
Initial body weight (IW)(g)	0.14 ± 0.04	0.14 ± 0.04	0.14 ± 0.04	0.14 ± 0.04
Final body weight (FW) (g)	0.45 ± 0.03^b^	0.55 ± 0.06^b^	0.56 ± 0.03^ab^	0.70 ± 0.04^a^
Body weight gain (WG) (g)	0.31 ± 0.003^b^	0.41 ± 0.06^b^	0.42 ± 0.03^b^	0.56 ± 0.03^a^
% WG	22.61 ± 0.23^b^	29.54 ± 4.52^b^	30.44 ± 1.68^b^	40.43 ± 1.98^a^
Initial length (cm)	2.13 ± 0.06	2.13 ± 0.06	2.13 ± 0.06	2.13 ± 0.06
Final length (cm)	3.16 ± 0.1^b^	3.36 ± 0.08 ^ab^	3.23 ± 0.03^ab^	3.63 ± 0.03^a^
length gain (cm)	0.97 ± 0.03^c^	1.1 ± 0.03^bc^	1.26 ± 0.05^b^	1.50 ± 0.05^a^
Specific growth rate (SGR)(/day)	1.82 ± 0.03^c^	1.89 ± 0.01^b^	1.94 ± 0.03 ^b^	2.04 ± 0.01^a^
Condition Factor	1.44 ± 0.05 ^c^	1.46 ± 0.05^c^	1.56 ± 0.05 ^b^	1.65 ± 0.08^a^
Feed conversion rate (FCR)	1.35 ± 0.1 ^a^	1.31 ± 0.04^ab^	1.26 ± 0.02^b^	1.19 ± 0.02^c^
Feed Efficiency (%)	78.16^c^	81.34 ^b^	82.01 ^b^	86.68^a^
Survival rate (%)	87.67 ± 0.1^c^	88.11 ± 0.05^c^	90.16 ± 0.08^b^	92.62 ± 0.3^a^

Data are presented as mean ± SD. Values with distinct superscripts in the same row indicate significant differences (*P* < 0.05). Diets: MME0 (0%, control); MME1 (0.25%); MME2 (0.5%); MME3 (1%)

### Immune parameters

The addition of MME significantly influenced mucosal immune parameters. Alkaline phosphatase (ALP) activity was consistently higher in all MME-supplemented treatment groups compared to the control (*p* < 0.05). Mucosal total protein levels were notably increased in the MME2 and MME3 groups, with both showing significant differences from the control (*p* < 0.05). While the MME1 group exhibited numerically higher total protein levels compared to the control, this increase was not statistically significant (*p* > 0.05). Lysozyme activity also showed a marked enhancement in the MME2 and MME3 groups (*p* < 0.05), with the highest increase observed in the MME2 group (Table 4).

**Table 4:** Mucus biochemical factors of zebrafish fed diets with various levels of methanolic *T. molitor* extract (MME) for 8 weeks (*n* = 9)

Parameters	MME0	MME1	MME2	MME3
Mucosal total protein (mg·mL⁻¹)	1.51 ± 0.05 ^b^	2.19 ± 0.37^ab^	2.89 ± 0.02 ^a^	2.85 ± 0.04 ^a^
Mucosal lysozyme (U mL^−1^)	45.55 ± 3.33 ^c^	53.88 ± 2.66^bc^	61.22 ± 3.11 ^a^	57.55 ± 2.77^ab^
Alkaline phosphatase (U mL^−1^)	27.00 ± 0.45 ^c^	39.51 ± 0.59 ^b^	37.67 ± 0.91 ^b^	42.84 ± 4.13 ^a^

Data represent the mean of 9 fish and are presented as mean ± SD. Values with distinct superscripts in the same row indicate significant differences (*P* < 0.05). Diets: MME0 (0%, control); MME1 (0.25%); MME2 (0.5%); MME3 (1%)

### Serum biochemical factors

The findings concerning serum biochemical parameters are shown in Table 5. Among the serum immunological parameters, increases in TP, total Ig and albumin were observed in groups MME2 and MME3 compared to both the MME0 and MME1 groups (*p* < 0.05), while lysozyme showed a non-significant dose-dependent increase in MME-supplemented groups compared to the control. Regarding the biochemical parameters, a dose-dependent decrease was observed in glucose levels, whereas no significant differences were observed in total cholesterol (TC), triglyceride, and LDH levels (*p* > 0.05).

**Table 5:** Serum biochemical parameters of zebrafish fed diets with various levels of methanolic *T. molitor* extract (MME) for 8 weeks (*n* = 9)

Serum biochemical factors	MME0	MME1	MME2	MME3
TP (g dL ^−1^ )	3.75 ± 0.26^b^	3.25 ± 0.36^b^	4.10 ± 0.07^a^	4.05 ± 0.32^a^
Immonoglobuline (g dL ^−1^ )	0.29 ± 0.15^b^	0.13 ± 0.18^b^	0.54 ± 0.03^a^	0.42 ± 0.23^a^
Lysozyme (U mL ^−1^ )	56.11 ± 7.22 ^b^	82.22 ± 3.33^a^	99.95 ± 4.45^a^	102.77 ± 8.33^a^
Total cholesterol (g dL ^−1^ )	438.33 ± 15.05^a^	436.83 ± 11.83^a^	426.75 ± 12.91 ^a^	426.25 ± 11.25 ^a^
Glucose (mg dL ^−1^ )	33.73 ± 0.08^a^	27.07 ± 1.40 ^b^	25.42 ± 0.37 ^b^	19.49 ± 0.50^c^
Albumin (g dL ^−1^ )	1.62 ± 0.59 ^b^	1.74 ± 1.15 ^b^	2.70 ± 0.12 ^a^	2.74 ± 0.17 ^a^
Triglycerides (mg dL ^−1^ )	352.50 ± 11.50^a^	350.08 ± 10.75^a^	349.50 ± 15.16 ^a^	348.16 ± 13.50 ^a^
LDH (U mL ^−1^ )	46.52 ± 3.02 ^a^	48.33 ± 3.62 ^a^	48.33 ± 6.04 ^a^	44.50 ± 3.08^a^

Data represent the mean of 9 fish and are presented as mean ± SD. Values with distinct superscripts in the same row indicate significant differences (*P* < 0.05). Diets: MME0 (0%, control); MME1 (0.25%); MME2 (0.5%); MME3 (1%)

### The intestinal tissue

**Fig. 1:**
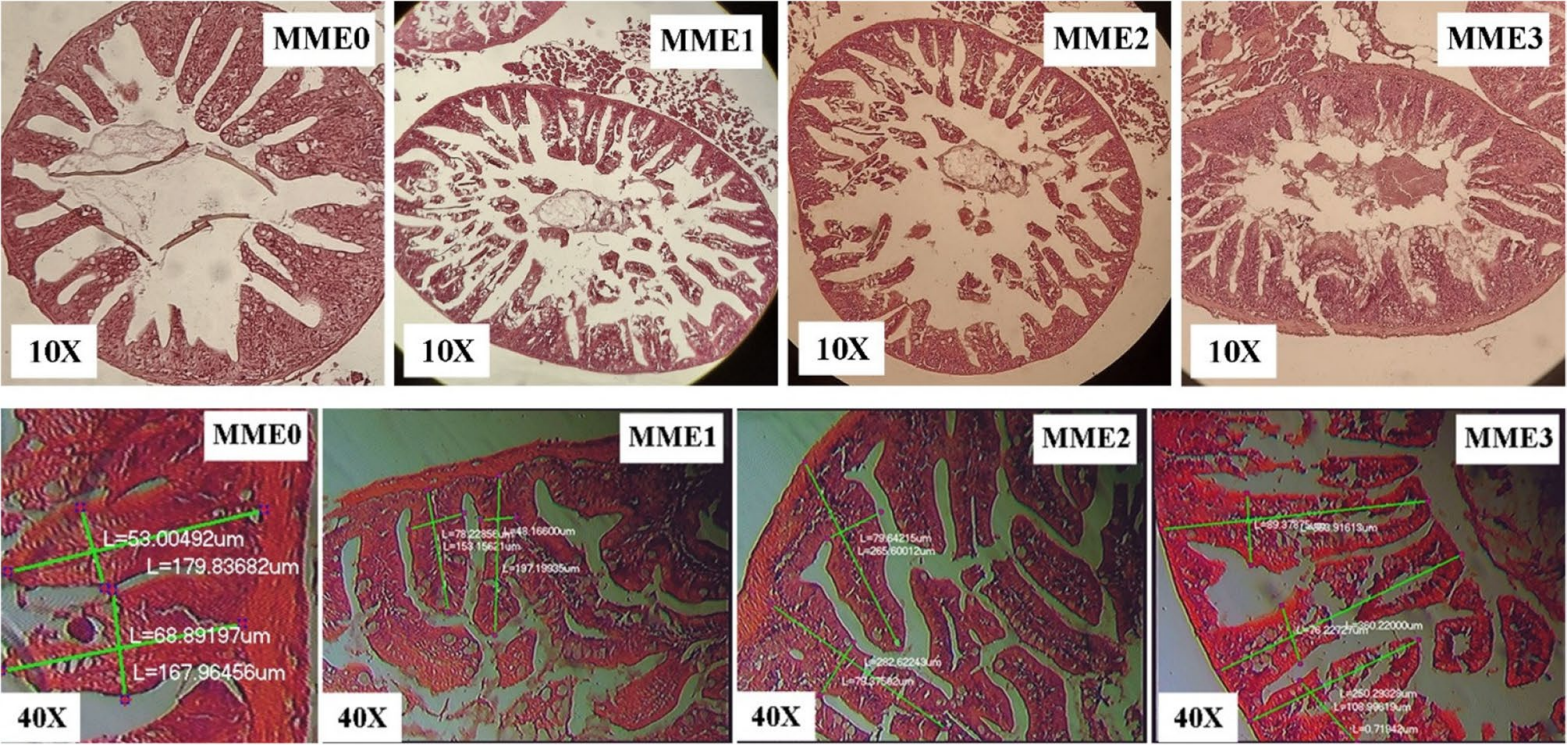
Photomicrograph of the intestine of zebrafish fed on different diets. Diets: MME0 (0%, control); MME1 (0.25%); MME2 (0.5%); MME3 (1%). Hematoxylin and eosin(H&E) stain

The villus height was found to be significantly higher only in group MME2, while the villus diameter was significantly higher only in group MME3 (*p* < 0.05), with no significant differences observed among the other groups (Table 6; Fig. 1).

**Table 6:** The intestinal villus height of zebrafish fed diets with various levels of methanolic *T. molitor* extract (MME) for 8 weeks (*n* = 9)

The intestinal villi	MME0	MME1	MME2	MME3
Villi height (µm)	164.49 ± 6.97^b^	153.19 ± 7.58^b^	259.52 ± 6.21^a^	254.25 ± 6.06^a^
Villi diameter (µm)	66.13 ± 4.80^b^	63.78 ± 7.53^b^	70.52 ± 7.01^b^	104.03 ± 8.54^a^

Data represent the mean of 9 fish and are presented as mean ± SD. Values with distinct superscripts in the same row indicate significant differences (*P* <0.05). Diets: MME0 (0%, control); MME1 (0.25%); MME2 (0.5%); MME3 (1%)

### Antioxidant enzymes

The SOD and CAT antioxidant enzyme activities did not show significant differences between control and MME groups, and also no significant differences among MME groups were observed (Table 7) (*p* > 0.05).

**Table 7:** SOD and CAT antioxidant enzyme activities of zebrafish fed diets with various levels of methanolic *T. molitor* extract (MME) for 8 weeks (*n* = 9)

Antioxidant enzymes	MME0	MME1	MME2	MME3
SOD (U mL^−1^)	6.16 ± 0.02 ^a^	6.15 ± 0.09^a^	6.14 ± 0.03^a^	6.15 ± 0.03 ^a^
CAT (U mL^−1^)	2.30 ± 0.01^a^	2.28 ± 0.04 ^a^	2.30 ± 0.02^a^	2.26 ± 0.005^a^

Data represent the mean of 9 fish and are presented as mean ± SD. Values with distinct superscripts in the same row indicate significant differences (*P* <0.05). Diets: MME0 (0%, control); MME1 (0.25%); MME2 (0.5%); MME3 (1%)

### Liver enzymes

The addition of mealworm led to an increase in ALP levels in groups MME2 and MME3 (*p* < 0.05), while it did not result in any changes in ALT and AST levels (Table 8) (*p* > 0.05).

**Table 8:** Liver enzymes of ALP, ALT, and AST of zebrafish fed diets with various levels of methanolic *T. molitor* extract (MME) for 8 weeks (*n* = 9)

Liver enzymes	MME0	MME1	MME2	MME3
ALP (U mL^−1^)	91.48 ± 5.50^b^	101.91 ± 0.73^ab^	105.53 ± 0.22^a^	108.98 ± 3.01^a^
ALT (U mL^−1^)	51.60 ± 3.30 ^a^	51.97 ± 4.33 ^a^	55.39 ± 1.65 ^a^	55.76 ± 2.68 ^a^
AST (U mL^−1^)	63.51 ± 5.29 ^a^	56.06 ± 3.63 ^a^	62.46 ± 2.17 ^a^	63.30 ± 0.48 ^a^

Data represent the mean of 9 fish and are presented as mean ± SD. Values with distinct superscripts in the same row indicate significant differences (*P* <0.05). Diets: MME0 (0%, control); MME1 (0.25%); MME2 (0.5%); MME3 (1%)

### Gene expressions

#### Immune-related gene expressions

**Fig. 2:**
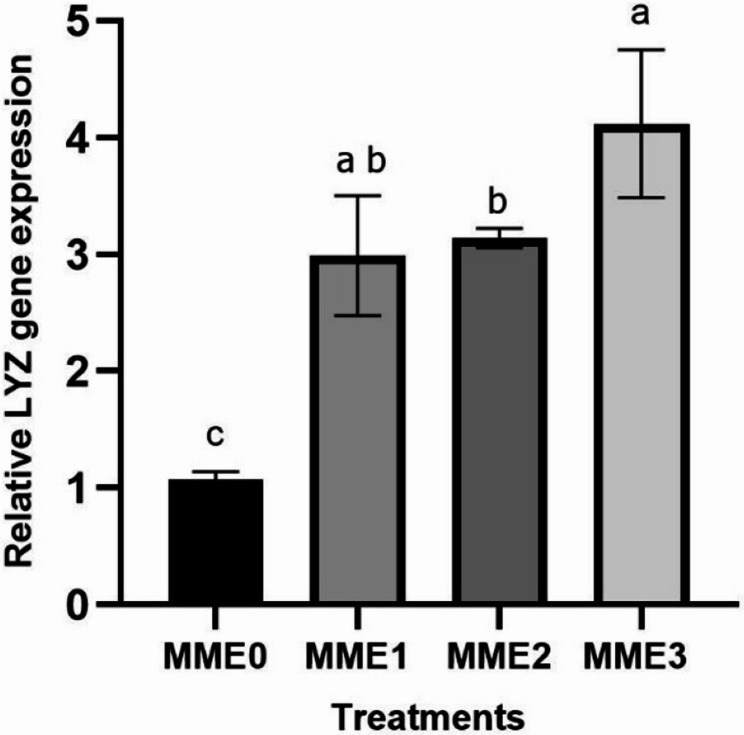
Relative expression levels lysozyme (*LYZ*) gene (fold change) in the intestine of zebrafish fed diets with various levels of methanolic *T. molitor* extract (MME) for 8 weeks (*n* = 9). Values are presented as mean ± SD. Different letters indicate significant difference among groups (*P* < 0.05). Diets: MME0 (0%, control); MME1 (0.25%); MME2 (0.5%); MME3 (1%)

**Fig. 3:**
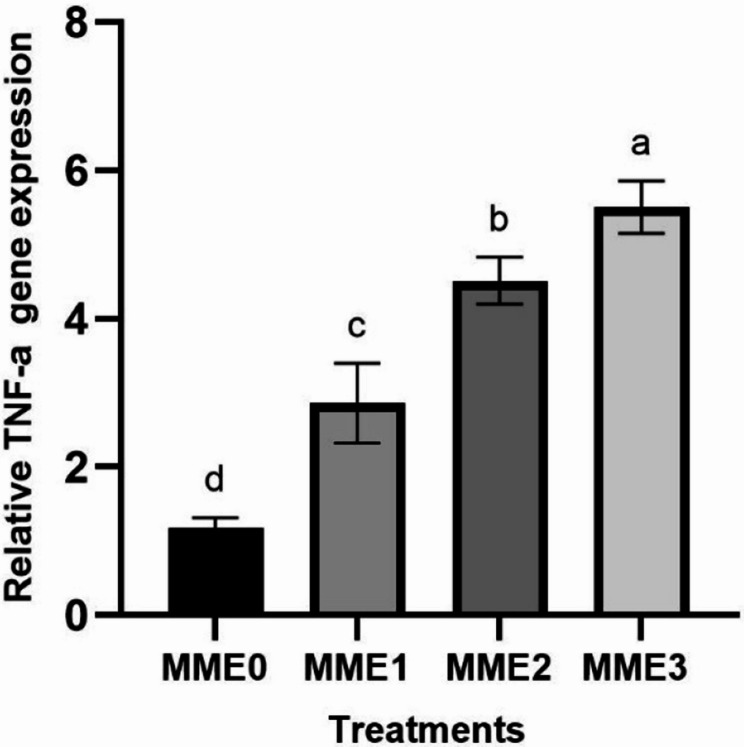
Relative expression levels of tumour necrosis factor-alpha (*TNF-α*) gene (fold change) in the intestine of zebrafish fed diets with various levels of methanolic *T. molitor* extract (MME) for 8 weeks (*n* = 9). Different letters indicate significant difference among groups (*P* < 0.05). Diets: MME0 (0%, control); MME1 (0.25%); MME2 (0.5%); MME3 (1%)

**Fig. 4:**
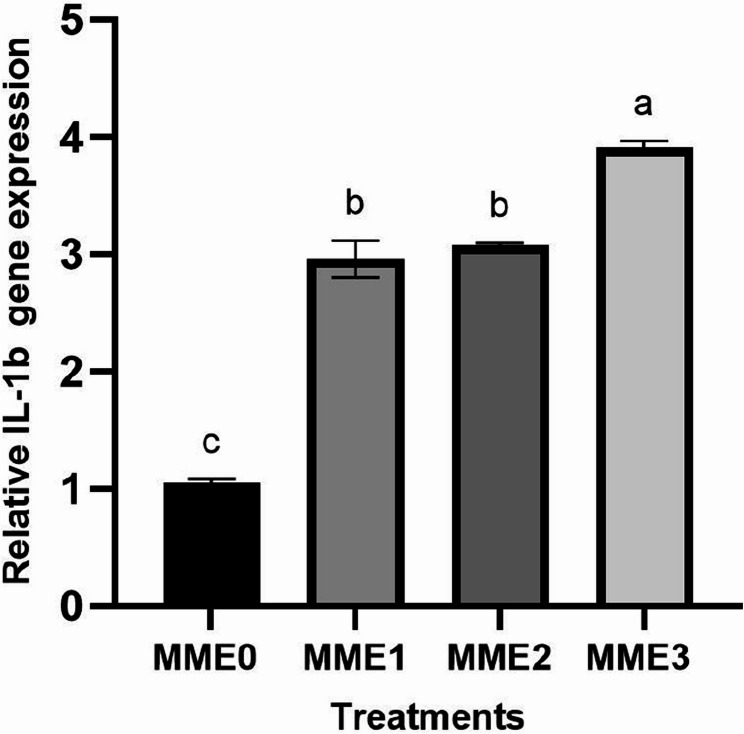
Relative expression levels of interleukin-1 (*IL-1*) gene (fold change) in the intestine of zebrafish fed diets with various levels of methanolic *T. molitor* extract (MME) for 8 weeks (*n* = 9). Values are presented as mean ± SD. Different letters indicate significant difference among groups (*P* < 0.05). Diets: MME0 (0%, control); MME1 (0.25%); MME2 (0.5%); MME3 (1%)

The expression levels of genes related to the immune system have exhibited significant increases across all levels with the addition of Methanolic mealworm extract. The highest increase in *LYZ* expression was observed at MME3 (3.92-fold) (*p* < 0.05), with no significant difference observed between MME1 (3-fold) and MME2 (3.14-fold) (Fig. 2). Similarly, *TNF-α* exhibited the highest increase at MME2 (4.51-fold) and MME3 (5.50-fold) groups, with MME1 (2.86-fold) showing a larger increase compared to the control but lower than MME2 and MME3 (Fig. 3) (*p* < 0.05). *IL-1* gene expression levels also demonstrated a dose-dependent increase. While MME1 and MME2 showed higher expression levels compared to the control, they were lower than those of MME3 (3.91-fold) (*p* < 0.05). No significant difference was observed between MME1 (2.96-fold) and MME2 (3.25-fold) (Fig. 4).

#### Antioxidant-related gene expressions

**Fig. 5:**
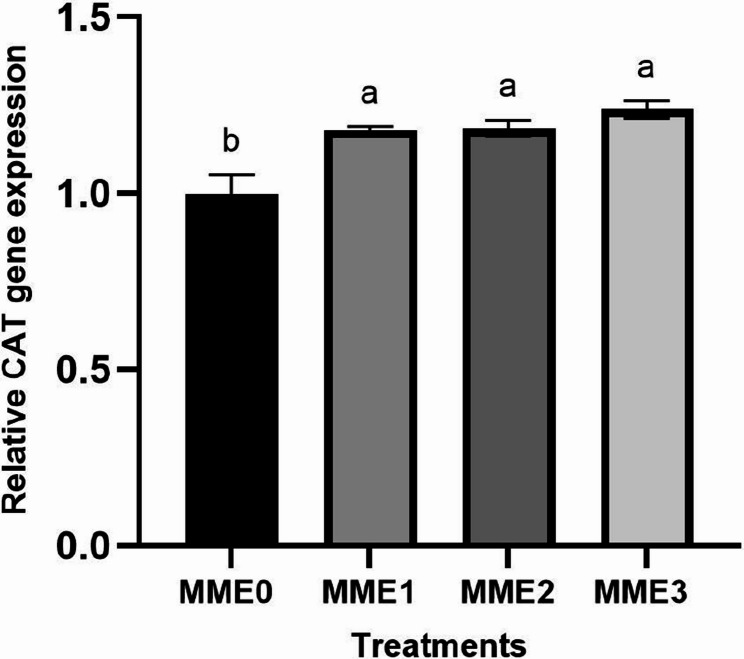
Relative expression levels of Catalase (*CAT*) gene (fold change) in the liver of zebrafish fed diets with various levels of methanolic *T. molitor* extract (MME) for 8 weeks (*n* = 9). Values are presented as mean ± SD. Different letters indicate significant difference among groups (*P* < 0.05). Diets: MME0 (0%, control); MME1 (0.25%); MME2 (0.5%); MME3 (1%)

**Fig. 6:**
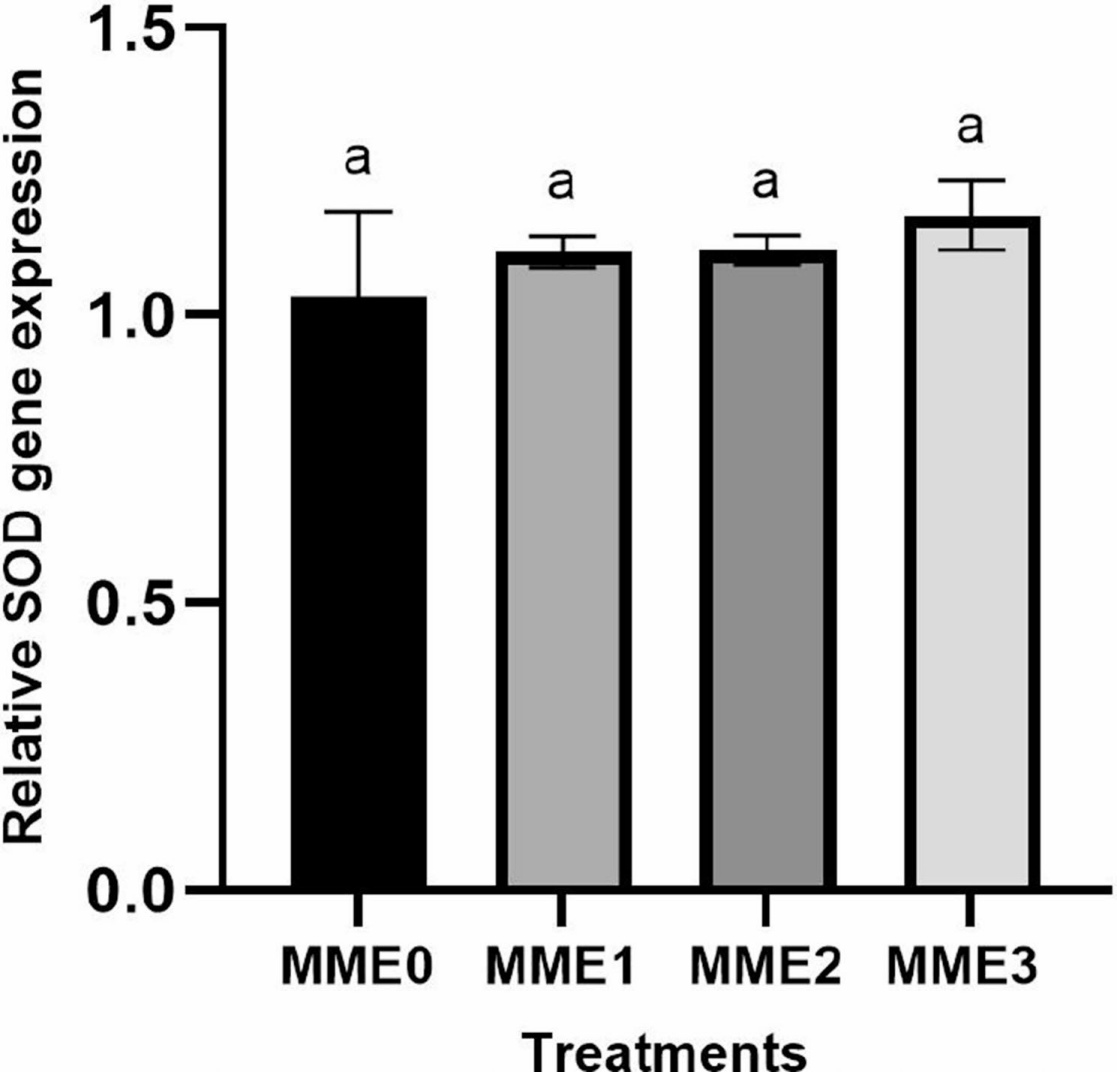
Relative expression levels of superoxide dismutase (SOD) gene (fold change) in the liver of zebrafish fed diets with various levels of methanolic *T. molitor* extract (MME) for 8 weeks (*n* = 9). Values are presented as mean ± SD. Different letters indicate significant difference among groups (*P* < 0.05). Diets: MME0 (0%, control); MME1 (0.25%); MME2 (0.5%); MME3 (1%)

The addition of mealworm did not result in any significant alterations in the expression levels of genes related to antioxidants, specifically catalase (*CAT*). And superoxide dismutase (*SOD*) (Figs. 5 and 6) (*p* > 0.05).

#### Growth and appetite-related gene expressions

**Fig. 7:**
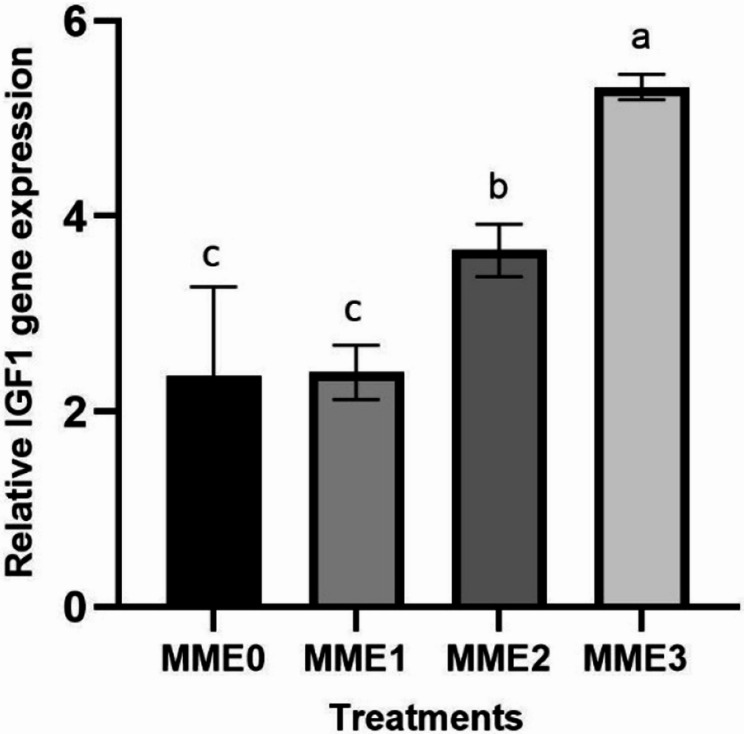
Relative expression levels of growth factor (*IGF-1*) gene (fold change) in the liver of zebrafish fed diets with various levels of methanolic *T. molitor* extract (MME) for 8 weeks (*n* = 9). Values are presented as mean ± SD. Different letters indicate significant difference among groups (*P* < 0.05). Diets: MME0 (0%, control); MME1 (0.25%); MME2 (0.5%); MME3 (1%)

**Fig. 8:**
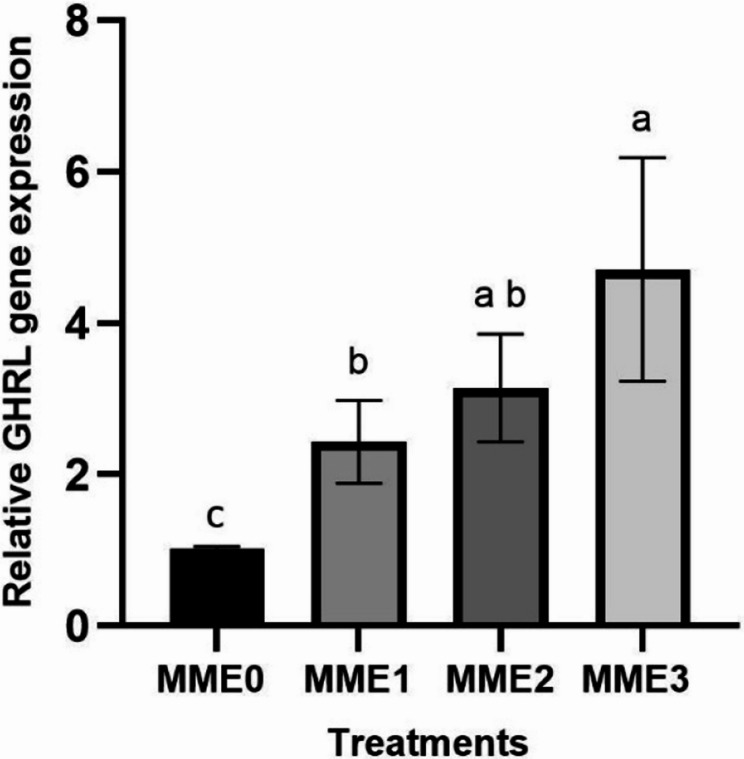
Relative expression levels of ghrelin (*GHRL*) gene (fold change), respectively, in the intestine of zebrafish fed diets with various levels of methanolic *T. molitor* extract (MME) for 8 weeks (*n* = 9). The data is presented as mean ± SD. Different letters indicate significant difference among groups (*P* < 0.05)*.* Diets: MME0 (0%, control); MME1 (0.25%); MME2 (0.5%); MME3 (1%)

**Fig. 9:**
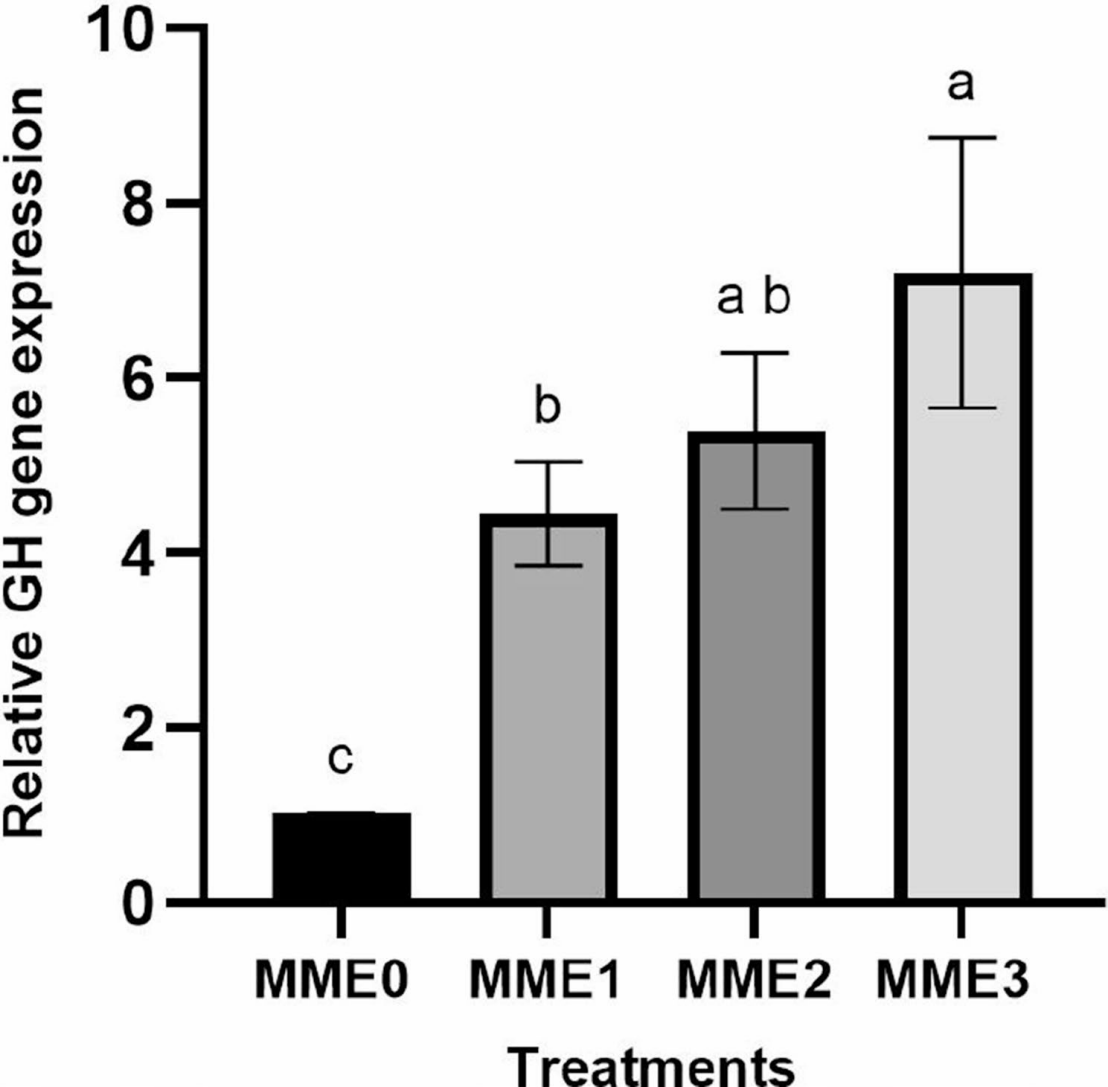
Relative expression levels of growth hormone (*GH*) gene (fold change) in the brain of zebrafish fed diets with various levels of methanolic *T. molitor* extract (MME) for 8 weeks (*n* = 9). The data is presented as mean ± SD. Different letters indicate significant difference among groups (*P* < 0.05). Diets: MME0 (0%, control); MME1 (0.25%); MME2 (0.5%); MME3 (1%).

The increased dosage of MME revealed a corresponding elevation in gene expression levels of growth-associated genes, notably *IGF1* (Fig. 7) (*p* < 0.05). Conversely, the highest increase in appetite-related gene (*GHRL*) (Fig. 8) and other investigated growth-related genes, such as *GH* (Fig. 9), was observed in the MME3 group (*p* < 0.05), with no significant difference between MME2 and MME1, yet yielding considerably higher results compared to the control.

.

.

## Discussion

Aquaculture is focused on ensuring food security and delivering high-quality products. To achieve this goal, there have been numerous endeavours in the aquaculture sector to introduce novel growth-promoting and maintaining health dietary supplements sourced from diverse origins into the diets of farmed aquatic organisms [1, 4, 38]. These efforts aim to enhance nutrient absorption, accelerate growth rates, and increase survival probabilities, ultimately strengthening the aquaculture industry [2, 13]. The products that were extracted and utilized in this research may be preferred over raw materials due to their significantly lower usage, as they minimize the risk of rejection by fish and facilitate the easier utilization of existing functional properties by fish. To our knowledge, this study represents the first attempt to investigate the effects of the methanolic extract of mealworms on growth, skin mucus and serum immune function, antioxidant defence, intestinal health and several gene expressions in zebrafish. Previous studies have focused mainly on full-fat or defatted mealworm meal in various fish species, with limited attention to solvent-extracted fractions and their specific bioactive effects.

### Growth performance and related gene expressions

This study provides novel evidence that dietary supplementation with methanolic mealworm extract (MME) positively influences growth performance and feed utilization in zebrafish. In particular, the MME3 group exhibited significant improvements—up to 80%—in parameters such as weight gain, length gain, condition factor, feed conversion ratio (FCR), survival rate, specific growth rate (SGR), and feed efficiency. These results suggest that high doses of mealworm extract may substantially enhance growth-related metrics in zebrafish.

Improving FCR is especially critical in aquaculture to reduce feed costs and increase sustainability by maximizing yield per unit of feed [7]. The dose-dependent improvements observed in FCR and SGR in this study support the hypothesis that bioactive compounds derived from mealworms may enhance nutrient utilization efficiency.

Although there are no directly comparable studies involving methanolic mealworm extract, similar growth-enhancing effects have been reported in various aquatic species using raw mealworm meal. For instance, positive growth performance has been observed in mandarin fish (*Siniperca scherzeri*) [33], giant freshwater prawn (*Macrobrachium rosenbergii*) [8], narrow-clawed crayfish (*Pontastacus leptodactylus*) (Mazlum et al., 2021), and largemouth bass (*Micropterus salmoides*) [6]. However, Gu et al. (2022) reported that while a 4% inclusion of mealworm meal did not adversely affect growth, higher inclusion levels led to reduced performance.

In this context, the observed improvements in growth performance in our study may be associated with changes in the expression of growth-related genes. MME supplementation notably upregulated the expression of *IGF-1* (Insulin-like Growth Factor 1) and *GH* (Growth Hormone), which are known to play critical roles in growth and development in fish. The strongest gene expression and growth responses were both recorded in the MME3 group. Additionally, *GHRL* (*Ghrelin*), a gene involved in appetite regulation, was also highly expressed in this group.

Ghrelin is a key peptide hormone that not only stimulates appetite but also triggers growth hormone release and plays a role in glucose homeostasis. The elevated *GHRL* expression observed in the MME3 group may have facilitated increased feed intake, thereby contributing to the observed growth enhancement.

These results align with previous studies that reported beneficial effects of insect-based feeds on fish growth and gene expression (Acar et al., 2021; Lin et al., 2023). For example, Acar et al. (2021) reported that moderate inclusion levels (25–50%) of insect-based protein blends containing *Tenebrio molitor* and *Hermetia illucens* significantly upregulated *GH* and *IGF-1* expression in rainbow trout muscle. In contrast, higher levels (75 and 100%) led to downregulation. the other hand, Li et al. [21] noted no significant change in *IGF-1* expression at moderate inclusion levels but reported downregulation at higher dosages.

Overall, this study highlights the potential of methanolic mealworm extract (MME) as a sustainable and cost-effective functional feed additive that enhances growth and feed utilization in zebrafish, likely through upregulation of growth-related genes. The results demonstrated a strong correlation between growth gene expression and weight gain data (*r* = 0.835, sig = 0.001). While zebrafish serve as a valuable model for nutritional and molecular research, caution is warranted in extrapolating these findings to other aquaculture species due to interspecies differences in digestive physiology and metabolism. Notably, the use of extract rather than whole insect meal allows for better isolation of bioactive compounds while minimizing indigestible components like chitin. However, the mechanisms underlying MME’s effects remain to be fully clarified. Therefore, species-specific optimization and long-term trials in commercially important fish are essential before widespread application in aquafeeds.

### Immunoregulation and metabolic stability

Fish skin mucus, rich in antimicrobial components, is recognized as a valuable biomarker for assessing immune response and overall health (Hoseinifar et al., 2019). In the present study, inclusion of methanolic mealworm extract (MME) in zebrafish diets significantly enhanced mucosal immunity markers—total protein (TP), lysozyme, and alkaline phosphatase (ALP)—with peak TP and lysozyme levels in the MME2 and MME3 groups, and notably elevated ALP activity in MME3. Lysozyme, which degrades both Gram-positive and Gram-negative bacteria and activates complement and phagocytosis, serves as a reliable indicator of immune modulation in fish [30]. Increases in mucosal TP also reflect improved general health, stress resilience, and nutritional status. Additionally, ALP’s antimicrobial activity and role in tissue repair further support its interpretation as a marker of improved mucosal immunity (Ahmadifar et al., 2019).

Serum immune markers also responded positively to supplementation. Plasma/serum biochemical parameters are considered highly sensitive indicators of physiological and health status in fish (Jeong et al., 2021; Sankian et al., [33]. MME2 and MME3 groups exhibited increased serum TP and total immunoglobulin (Ig) compared to the control and MME1. This aligns with findings in Atlantic salmon (*Salmo salar*), where mealworm inclusion raised Ig levels without altering TP (HabteTsion et al., 2024). All MME-treated groups additionally showed elevated serum lysozyme consistent with studies in various species [8, 33]; Zhang et al., 2022), though effects vary by species [12, 25]. Taken together, these results suggest that mealworm extract can enhance both mucosal and systemic immunity, potentially via antimicrobial peptides or chitin-mediated immune stimulation [18].

In assessing metabolic impact, dose-dependent reductions in glucose were observed, while total cholesterol (TC), albumin, triglycerides, and LDH remained stable. Stress in fish activates the HPA axis, increasing cortisol and catecholamines, which drives gluconeogenesis and glycogenolysis to elevate plasma glucose, an adaptation to stress (Ray & Sinha, 2014). A reduction in glucose, therefore, indirectly suggests a modulated stress response, further supported by stable LDH activity, despite cortisol not being directly measured [25]. Albumin and triglyceride levels stayed within normal ranges, indicating that MME does not impair hepatic protein synthesis or lipid metabolism, nor provoke hyperlipidemia or energy storage disruptions (Aski et al., 2022; Jeong et al., 2021; Javed et al., 2017). Collectively, these findings affirm the metabolic safety of MME at the tested doses. Nevertheless, we recognize that the possibility of type II errors can never be excluded and might have been responsible for these findings.

Effects of insect-based diets, including mealworm, on lipid metabolism vary across species. For example, dietary insect inclusion reduced plasma lipids in mandarin fish and lipids in Jian carp and European sea bass [20, 33]. In rainbow trout, mealworm inclusion led to dose-dependent cholesterol reduction [18], while other studies found no effect (Khosravi et al., 2018; Mamuad et al., 2021). Such discrepancies likely reflect differences in species, size, diet composition, and insect quality (Jeong et al., 2021; Su et al., 2017). Chitin, abundant in insect exoskeletons, may underlie lipid modulation by limiting lipid absorption at high doses, though at moderate levels it may act as a prebiotic and immune enhancer (Belforti et al., 2015; Henry et al., [12].

Liver enzyme activities (ALT, AST, ALP) further corroborated liver safety: no significant changes occurred in ALT or AST, consistent with prior mealworm feeding studies in various fish species (Khosravi et al., 2018). The increase in ALP, especially in MME3, aligned with enhanced growth and upregulated growth-associated genes (GH, IGF1), suggesting a physiological rather than pathological response (Habte Tsion et al., 2024). However, the absence of histological analysis means potential subclinical changes cannot be ruled out, thus warranting histopathological evaluation in future studies.

Gene expression analysis further supported the macroscopic and biochemical findings. In the present study, dietary supplementation with methanolic mealworm extract (MME) led to a dose-dependent upregulation of key immune-related genes, particularly *LYZ*, *TNF*, and *IL-1*, with the highest expression observed in the MME3 group. This suggests an enhanced immune activation at higher inclusion levels. These results are consistent with previous studies that reported increased expression of cytokines and antimicrobial genes in fish fed mealworm- or other insect-derived diets [6]; Zhang et al., 2024; Su et al., 2017; Acar et al., 2021). The immunostimulatory effects are likely attributed to bioactive compounds such as chitin and tenecin-1 present in insect biomass. However, it is important to note that excessive inclusion of insect meals may lead to overstimulation of the immune system. For instance, Ge et al. [9, 43] reported that high levels of mealworm inclusion could elevate pro-inflammatory cytokine expression (e.g., *IL-1β*, *TNF-α*) and induce intestinal inflammation, potentially impairing nutrient absorption and overall gut health. Therefore, while the observed immunostimulation is promising, the formulation of insect-based diets should be carefully optimized to balance efficacy and safety.

The findings indicate that zebrafish fed MME-supplemented diets exhibit enhanced immune responses, improved metabolic stability, and preserved liver function. The immunostimulatory effects (e.g., heightened TP, lysozyme, ALP, and immune gene expression) and metabolic safety profile (e.g., reduced glucose, stable albumin, triglycerides, and liver enzymes) suggest that MME is a promising functional feed ingredient. However, without histological analyses, interpretations of ALP elevation remain tentative. While zebrafish is a valuable model for aquatic vertebrates, confirming MME’s efficacy and safety in other fish species, different life stages, and long-term feeding trials is essential. Future studies incorporating hormonal profiling, histopathology, and extended metabolic assessments will be critical to evaluate mealworm-derived additives for aquaculture applications fully.

### Antioxidant enzyme activities and gene expression

Oxidative stress arises when the antioxidant defence system fails to adequately neutralize reactive oxygen species (ROS), resulting in cellular damage and metabolic dysfunctions in fish [20]. Among enzymatic defences, superoxide dismutase (SOD) and catalase (CAT) form a primary line of protection: SOD converts superoxide anions into hydrogen peroxide, which is subsequently degraded by CAT to water and oxygen (Zhang et al., 2024). These enzymes are often used as biomarkers to evaluate oxidative balance and overall health in fish (Jeong et al., 2021; Li et al., 2022).

Despite previous evidence demonstrating that various insect meals can upregulate antioxidant enzyme activities—thereby mitigating oxidative damage and enhancing radical scavenging capacity in fish (Jeong et al., 2021; Su et al., 2017)—our findings reveal that methanolic mealworm extract (MME), under the tested conditions, failed to induce any significant changes in both SOD and CAT enzyme activities or the expression of their related genes. This suggests that, under the tested conditions, MME did not activate the antioxidant defence system at either the biochemical or molecular levels. The lack of antioxidant stimulation may reflect a low oxidative burden in the fish or insufficient stimulation by MME at the dosages applied.

This finding is consistent with several previous studies. For example, Sankian et al. [33] reported that the inclusion of 10–30% mealworm meal did not alter SOD activity in mandarin fish (*Siniperca scherzeri*), although GPX activity was increased. Similarly, Li et al. (2022) showed that adding mealworm alongside soybean meal in carp diets did not influence CAT mRNA expression, although they noted increased SOD activity. In contrast, Melenchón et al. (2021) and Chen et al. [6] found that insect meal inclusion in rainbow trout and largemouth bass diets led to increased activities and expression of antioxidant enzymes, but these effects were dose-dependent and occasionally reversed at higher inclusion levels.

The inconsistencies in antioxidant responses may be attributed to several factors, including fish species, developmental stage, health status, dietary composition, and the physical-chemical nature of the insect supplement (e.g., raw, defatted, extract). Insect-derived components such as chitin and its derivatives (e.g., chitosan) have been suggested to confer antioxidant properties through radical scavenging mechanisms [6], but these effects may only manifest at specific inclusion levels or in specific host contexts. In our study, neither biochemical enzyme activity nor gene expression supported such a mechanism, indicating that MME under the applied conditions had limited antioxidant efficacy.

Moreover, the current analysis was limited to SOD and CAT. The omission of additional oxidative stress markers such as GPX, MDA, and T-AOC represents a constraint in evaluating the complete antioxidant profile. As suggested by Zhang et al. (2024), including a broader range of markers, along with histopathological evaluations, would offer a more comprehensive understanding of antioxidant modulation in response to insect-derived feed additives. While chitin and its derivatives—naturally occurring in insect-based products—are known for their potential free radical scavenging properties, their specific role in mediating antioxidant responses under the present experimental conditions remains to be clarified. Future studies may consider these aspects to define better the antioxidant potential of methanolic insect extracts in aquaculture.

Additionally, the process of methanolic extraction, while effective in concentrating specific bioactive compounds, requires the use of organic solvents and additional energy inputs, which may raise concerns regarding environmental sustainability. In contrast, the production of insect biomass, including mealworm, often utilizes organic waste substrates, thereby contributing to circular bioeconomy practices. Therefore, future assessments should include a life-cycle perspective, balancing extraction efficiency with ecological and economic trade-offs.

### Histology

#### The intestinal villi

The intestine plays a central role in nutrient digestion and absorption, and its structural integrity is critical for maintaining overall physiological homeostasis in fish [20]. The morphology of intestinal microvilli can be modulated by dietary protein sources, prompting various histological studies in aquaculture to investigate these effects [6]. In this regard, many insect species, such as the yellow mealworm (YM), have been reported to provide effective resistance against pathogenic bacteria and exert beneficial effects on animal intestinal health and immunity [9].

In our study, the villus height was found to be significantly higher only in group MME2. In contrast, the villus diameter was significantly higher only in group MME3, with no significant differences observed among the other groups.

## Conclusion

This study demonstrates the potential of methanolic mealworm extract as a dietary supplement in aquaculture, particularly for zebrafish. The inclusion of mealworm extract positively impacted growth performance, feed efficiency, and immune responses, especially at the highest dosage (MME3). Although higher inclusion levels of mealworm meal have been reported to cause adverse effects in other species, our findings suggest that mealworm extract was well tolerated in zebrafish and did not result in observable negative impacts under the present experimental conditions. Furthermore, while the extract did not significantly alter antioxidant gene expression, it did not negatively affect fish health, as indicated by stable biochemical parameters and liver enzyme activities. The observed benefits, such as improved growth-related gene expression, highlight the promise of mealworm extract in enhancing the sustainability and efficiency of aquaculture practices. While the present findings in zebrafish are promising, they should not be generalized to other aquaculture species without further validation. Future research should focus on optimizing inclusion rates and evaluating the long-term effects on different fish species to fully realize the potential of mealworm-based feeds in the aquaculture industry.

## Data Availability

The data that support the findings of this study are available upon reasonable request to the corresponding authors.
